# Mechanisms Regulating GLUT4 Transcription in Skeletal Muscle Cells Are Highly Conserved across Vertebrates

**DOI:** 10.1371/journal.pone.0080628

**Published:** 2013-11-18

**Authors:** Rubén Marín-Juez, Mónica Diaz, Jordi Morata, Josep V. Planas

**Affiliations:** Departament de Fisiologia i Immunologia, Facultat de Biologia, Universitat de Barcelona and Institut de Biomedicina de la Universitat de Barcelona (IBUB), Barcelona, Spain; Tohoku University, Japan

## Abstract

The glucose transporter 4 (GLUT4) plays a key role in glucose uptake in insulin target tissues. This transporter has been extensively studied in many species in terms of its function, expression and cellular traffic and complex mechanisms are involved in its regulation at many different levels. However, studies investigating the transcription of the GLUT4 gene and its regulation are scarce. In this study, we have identified the GLUT4 gene in a teleost fish, the Fugu (*Takifugu rubripes*), and have cloned and characterized a functional promoter of this gene for the first time in a non-mammalian vertebrate. *In silico* analysis of the Fugu GLUT4 promoter identified potential binding sites for transcription factors such as SP1, C/EBP, MEF2, KLF, SREBP-1c and GC-boxes, as well as a CpG island, but failed to identify a TATA box. In vitro analysis revealed three transcription start sites, with the main residing 307 bp upstream of the ATG codon. Deletion analysis determined that the core promoter was located between nucleotides -132/+94. By transfecting a variety of 5´deletion constructs into L6 muscle cells we have determined that Fugu GLUT4 promoter transcription is regulated by insulin, PG-J2, a PPARγ agonist, and electrical pulse stimulation. Furthermore, our results suggest the implication of motifs such as PPARγ/RXR and HIF-1α in the regulation of Fugu GLUT4 promoter activity by PPARγ and contractile activity, respectively. These data suggest that the characteristics and regulation of the GLUT4 promoter have been remarkably conserved during the evolution from fish to mammals, further evidencing the important role of GLUT4 in metabolic regulation in vertebrates.

## Introduction

In mammals, the glucose transporter 4 (GLUT4) is the main facilitative glucose carrier responsible for the insulin-regulated glucose uptake in skeletal muscle and adipose tissue [1]. GLUT4 exerts its function by translocating to the plasma membrane from intracellular stores in response to insulin [2] but also in response to muscle contraction [3], allowing the entry of glucose into muscle cells. Because of this, GLUT4 has been described as the main glucose transporter responsible for insulin-mediated glucose uptake in muscle contributing to systemic glucose uptake in postprandial conditions. 

The regulation of the expression of the GLUT4 gene is governed by complex mechanisms as it is subjected to both tissue-specific and hormonal metabolic regulation [4]. Changes in GLUT4 expression are observed in physiological states of altered glucose homeostasis. GLUT4 mRNA levels in skeletal muscle increase with exercise training and decrease during states of insulin deficiency [5,6,7] due to alterations in the transcription rate of the GLUT4 gene [8,9]. Therefore, unraveling the mechanisms involved in the regulation of GLUT4 transcription will assist in understanding the molecular processes regulating glucose homeostasis. In this light, previous studies have characterized several cis-acting elements regulating the transcription of the human, mouse and rat GLUT4 promoters using transgenic mice models. It has been reported that a region of 1154 bp of the 5´-flanking region of the human GLUT4 gene is essential to regulate its expression in response to insulin [10]. The regions located within 730 bp upstream of the human GLUT4 gene [10] and 522 bp upstream of the rat GLUT4 gene [11] contain motifs shown to be essential for the tissue specific expression of the GLUT4 promoter, such as binding sites for the myocyte enhancer factor 2 (MEF2). Other factors that appear to be involved in the transcriptional regulation of the GLUT4 gene include SP1, CCAAT/enhancer-binding protein (C/EBP), peroxisome proliferator-activated receptor-γ (PPARγ), hypoxia inducible factor 1α (HIF-1α), E-box, sterol regulatory element binding protein 1c (SREBP-1c), Krüppel-like factor 15 (Klf15) and nuclear factor NF1 [12,13]. Strikingly, little is known regarding the transcriptional activation or regulation of the GLUT4 gene in mammals and to date there is no information in lower vertebrates. Current evidence indicates that GLUT4 mRNA levels in skeletal muscle of teleost fish are increased by hormonal stimuli (i.e. insulin and IGF-1) [14,15], swimming-induced activity [16] and activators of AMP-activated protein kinase [17], suggesting that GLUT4 may also play an important role in the regulation of glucose homeostasis in lower vertebrates. In this light, for a better understanding of the regulation of the GLUT4 gene in lower vertebrates, we set out to identify and functionally characterize the GLUT4 promoter in Fugu (*Takifugu rubripes*), a teleost fish. Fugu was chosen as a model organism for this study because it was the second vertebrate genome to be sequenced, after the human genome, and its compact genome has proven to be a useful reference for the identification of genes and regulatory elements in other vertebrate genomes.

In this study, we report on the identification of the GLUT4 gene in Fugu and on the characterization of a functional promoter region of the Fugu GLUT4 gene. We have identified three transcription start sites (TSSs) and demonstrated the homology of this regulatory region with that of the GLUT4 gene in other teleost species by *in silico* analysis. Moreover, we show that the regulatory region of the Fugu GLUT4 gene presents most of the binding motifs described as important for the transcriptional regulation of GLUT4 in mammals. In addition, we provide evidence that the transcription of the Fugu GLUT4 gene in skeletal muscle cells is inhibited by insulin and stimulated in response to PG-J2 (a PPARγ agonist) and to electrical stimulation. Transient transfection of various 5´deletion constructs showed that the response to PG-J2 depends on the number of PPARγ binding motifs and suggests the implication of HIF-1α in the regulation of the transcriptional response of the Fugu GLUT4 gene to contractile activity. 

## Materials and Methods

### Materials

Human recombinant insulin and 15-deoxy-Δ^12,14^-prostaglandin J2 (PG-J2) were purchased from Sigma (St Louis, MO, USA). α-MEM, fetal bovine serum (FBS) and other tissue culture reagents were purchased from Invitrogen (Prat del Llobregat, Spain).

### Cloning and sequence analysis of the Fugu GLUT4 gene and its promoter

The genomic sequence corresponding to the Fugu GLUT4 (*slc2a4*) gene was identified by searching the Fugu genomic sequence database in Ensembl (scaffold_63:253869-258674:-1; www.ensembl.org). In order to clone the Fugu GLUT4 promoter region, we designed gene specific primers incorporating the KpnI and XhoI restriction sites (Table 1) to amplify a 1.3 kb genomic region upstream of the identified Fugu GLUT4 gene (scaffold_63:258623-259996). PCR using these primers and genomic DNA from Fugu as template (purchased as biological materials from the Medical Research Council Human Genome Mapping Project Resource Center, UK, now closed) was performed under the following conditions: 3 min at 94 °C followed by 35 cycles of 94 °C for 45 s, 59 °C for 30 s and 72 °C for 1 min, and a final step of 72°C for 10 min. The PCR product of 1314 bp was isolated from the agarose gel using Geneclean Spin Kit (MP Biomedicals, Solon, Ohio), cloned into the pGEM-T Easy vector (Promega, Madison, WI, USA) and sequenced in both strands with the BigDye v3.1 sequencing kit (Applied Biosystems, Foster City, CA). The Fugu GLUT4 promoter sequence was aligned to the available sequences of GLUT4 promoters from other teleost species (*Tetraodon*, *Tetraodon nigrovirus*; stickleback, *Gasterosteus aculeatus* and medaka, *Oryzias latipes*) in Ensembl and their conservation profiles were analyzed with zPicture software (zpicture.dcode.org [18]). Analysis of the GLUT4 promoter sequences for transcription factor (TF) binding sites was conducted with MatInspector (www.genomatix.com [19]) and AliBaba 2.1 softwares (www.gene-regulation.com/pub/programs/alibaba2; [20]). CpG island mapping was performed using MethPrimer software (www.urogene.org/methprimer; [21]). The gene order of the human GLUT4 locus in chromosome 17 and that of the Fugu GLUT4 locus in scaffold 63 was obtained from Ensembl release version 71.

**Table 1:** Primer sequences used in the cloning of the deletion constructs and in the identification of the transcription start sites.

**Primer Name**	**Usage**	**Primer sequence (5´→3´)**
-1072_For	-1072 Cloning	TTGCA**GGTACC**TTGTGCCGTGAGAGCGTCAATG
-841_For	-841 Cloning	TTGCA**GGTACC**TCAGATGGTGTCAAGTTCCTCCGTTC
-766_For	-766 Cloning	TTGCA**GGTACC**AAAGGAGGTGGCGTGATGTGGG
-608_For	-608 Cloning	TTGCA**GGTACC**CCTGTTGCCTGGTTGAAATGGAT
-324_For	-324 Cloning	TTGCA**GGTACC**TGGTCTCCACATTGGATTTGTTGAG
-132_For	-132 Cloning	TTGCA**GGTACC**TCTCAAGAACAGAGGCGCAGTGG
+94_For	+94 Cloning	TTGCA**GGTACC**TGCAGCTTGAGGTCGAGACTTGTT
pGLUT4_Rev	Cloning	TTGCA**CTCGAG**CAGGCAGCTGCAGATGACAGAT
GSP1_Rev	GeneRacer	CCCACGCAGAAGGAGGACAACAT
GSP2_Rev	GeneRacer	CAGAGATCCCAGGACGGCGGTGAAG

a The sequences of the KpnI and XhoI sites are indicated in bold.

b Sequences added to increase the efficiency of the digestion are indicated in italics.

### Generation of luciferase reporter gene constructs

The firefly (*Photinus pyralis*) luciferase pGL3 expression vector system (Promega) was used as a reporter system to evaluate the transcriptional activity of the 5´-flanking region of the Fugu GLUT4 gene. The 1.3 kb Fugu GLUT4 promoter fragment was excised from the pGEM-T Easy vector by digestion with KpnI and XhoI (New England Biolabs, MA, USA) and ligated into the pGL3-basic vector digested with the same restriction enzymes to allow transcription of the firefly luciferase gene under the control of the Fugu GLUT4 promoter (pGL3-FuguGLUT4P). Following the same strategy, a series of unidirectional 5′ deletion constructs were generated by PCR using specific primers with the sequence of the KpnI and XhoI restriction sites incorporated (Table 1) and pGL3-FuguGLUT4P as template. All the constructs were verified by sequencing at least two times from each side with RV3 and GL2 vector primers using the BigDye v3.1 sequencing kit (Applied Biosystems, Foster City, CA). A promoterless luciferase reporter vector, pGL3-Basic (Promega), was used in the course of these studies as a negative control. The pRL-TK expression plasmid, containing the cDNA encoding sea pansy (*Renilla reniformis*) luciferase under the control of the HSV-thymidine kinase promoter, was also used as an internal control for transfection efficiency (Promega). A reporter pGL3-Control vector (firefly luciferase gene under the control of the SV40 promoter) was used as a positive control of luciferase luminescence (Promega). A rat GLUT4 promoter construct (pGL3-ratGLUT4) containing a 2.2 kb DNA fragment upstream of the TSS of the rat GLUT4 gene, kindly donated by Dr. Rafael Salto (University of Granada, Granada, Spain) [22], was used as a positive control and for comparison of activity levels with pGL3-fuguGLUT4P. 

### Cell strains and cell culture conditions

The rodent-derived skeletal muscle cells lines L6 (rat; kindly donated by Dr. Amira Klip, The Hospital for Sick Children, Toronto, Canada; [23]) and C2C12 (mouse; purchased from ATCC, Barcelona, Spain) were maintained with α-MEM containing 10% FBS and 1% antibiotic-antimycotic solution (10,000 U/ml penicillin G, 10 mg/ml streptomycin, 25 μg/ml amphotericin B) in an atmosphere of 5% CO_2_ at 37 °C. At confluence, C2C12 myoblasts were induced to differentiate into myotubes by culturing the cells for at least 5 days in α-MEM with the percentage of FBS reduced to 2%.

### Transient transfections and *GLUT4* promoter activity measurements

Approximately 1 x 10^5^ L6 myoblasts per well were plated in 24-well dishes and 2.5 x 10^5^ C2C12 myoblasts were plated per well in 12-well dishes. Cells were cultured for 24 hours until 80-90% confluence and were transfected with the plasmids described above (1μg/well) using the Lipofectamine2000^TM^ reagent (Invitrogen), following the manufacturer’s indications. Briefly, cells were plated at 90 % confluence and cultured overnight with α-MEM containing 10 % FBS without antibiotics at 37 °C. Lipofectamine2000 reagent was mixed with α-MEM without FBS/antibiotics (4.5 μl/150 μl medium), incubated for 5 min at room temperature, combined with construct DNA (1.5 μg/150 μl for pGL3 and 0.5 μg/150 μl for pRL-TK) and further incubated for 20-30 min at room temperature. Cells were washed once with PBS and a total of 100 μl of DNA-Lipofectamine2000 reagent mixture was added to each well (in triplicate) and incubated for 2-4 h under 5 % CO_2_ at 37 °C. After this period, 250 μl of α-MEM-20 % FBS without antibiotics were added to each well. Cells were incubated for 24 h and observed for evidence of cytotoxicity under the microscope. After transfection, L6 myoblasts were cultured in the absence or presence of human recombinant insulin (1, 10 and 100 nM) or PG-J2 (10 μM) for 18 h. Time-course experiments were performed with L6 myoblasts transfected with the pGL3-FuguGLUT4P construct in the absence or presence of insulin (100 nM) for 0.5, 1, 2, 4, 8 and 18 h. Transiently transfected C2C12 myotubes plated in 12 well dishes were subjected to electrical pulse stimulation using an Electrical Stimulator of Cultured Cell System (ESCC), as described by Marotta and co-workers [24]. The electrical pulse stimulations were carried out inside a cell incubator for 90 min at 37 °C in an atmosphere of 5% CO_2_, at 40 V with a pulse duration of 30 ms and a frequency of 3 Hz. Control C2C12 myotubes were incubated in the absence of electrical pulse stimulation. After stimulation, L6 myoblasts and C2C12 myotubes were lysed and luciferase activity was analyzed using the Dual-Luciferase Reporter Assay System (Promega), according to the manufacturer’s instructions. Luciferase activity measurements were performed with an Infinite^®^ M200 reader (Tecan Trading AG, Switzerland). The level of firefly luciferase activity was normalized to that of sea pansy luciferase activity for each transfection.

### Determination of the TSS in the Fugu *GLUT4* gene

The TSS of Fugu GLUT4 gene was determined using the GeneRacer Kit (Invitrogen) following the manufacturer’s indications. This technique is based on RNA ligase-mediated rapid amplification of 5´and 3´cDNA ends (RLM-RACE) and oligo-capping rapid amplification cDNA ends (RACE) methods. Nested PCR was performed using gene specific primers (GSPs) (Table 1) and total RNA isolated from Fugu dorsal muscle kindly donated by Dr. Shugo Watabe (University of Tokyo) as template. Fragments were amplified using Platinum^®^ *Taq* DNA Polymerase (Invitrogen). PCR conditions were as follows: 94°C for 3 min followed by 35 cycles of 94°C for 45 s, 60°C for 30 s and 72°C for 1 min, and a final step of 72°C for 10 min. Fragments were ligated into pCR^®^4-TOPO (Invitrogen) and transformed into One Shot^®^ TOP10 competent cells (Invitrogen).

### Statistical analyses

Statistical analyses were performed using SPSS11 (SPSS, Chicago, IL). Statistical differences were analyzed by Kruskal–Wallis and Mann–Whitney non-parametric tests and were considered to be significant at *p* < 0.05.

## Results

### Identification and in silico analysis of the Fugu GLUT4 gene

**Figure 1:**
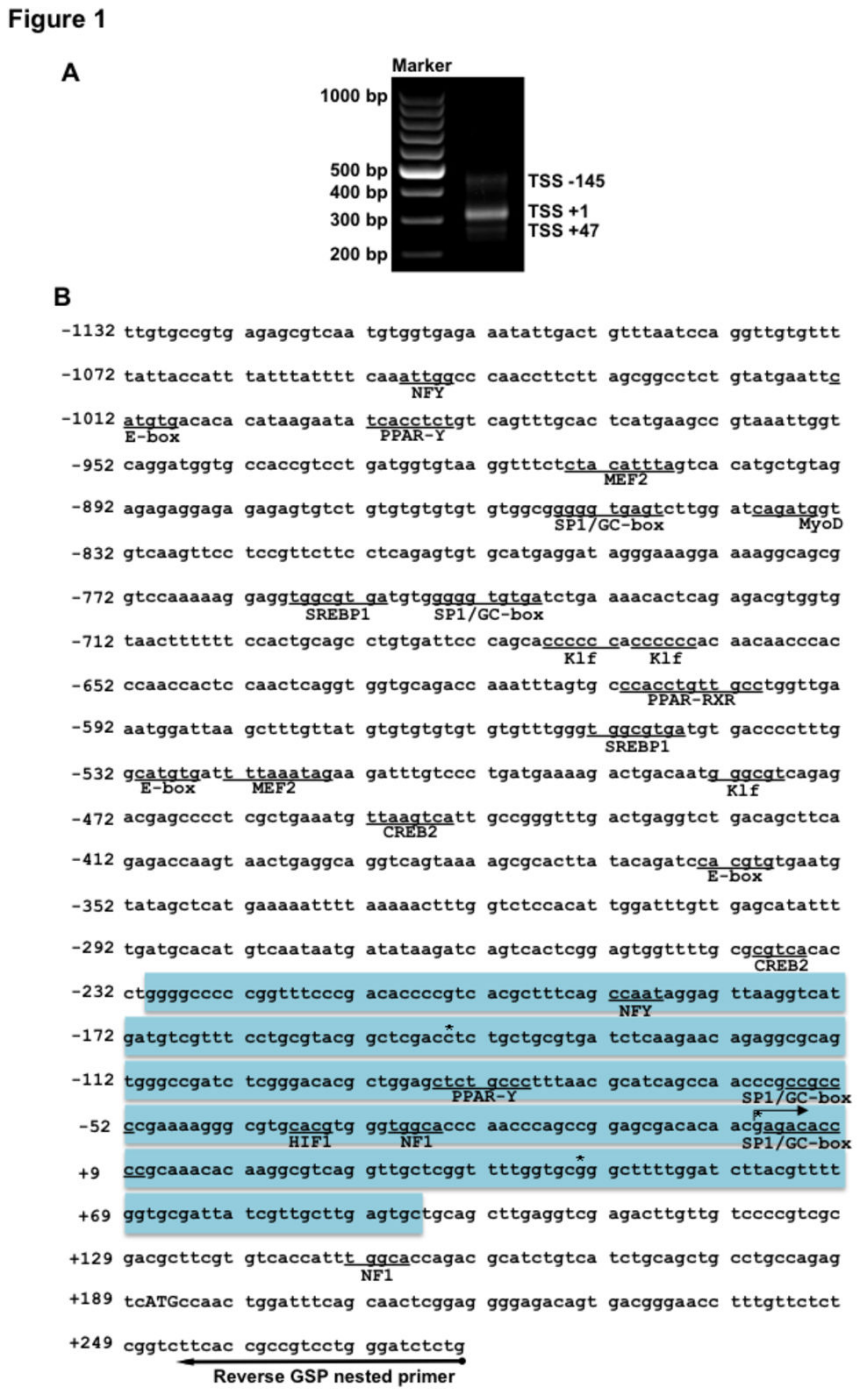
Analysis of the 5´ flanking region of the Fugu GLUT4 gene. (A) Determination of the transcription start sites (TSS) by 5´RACE. Agarose gel electrophoresis of nested PCR reaction products from 5´ RACE yielded three product sizes of 452, 307 and 261 bp and were denoted as -145, +1 and +47 respectively. (B) Sequence of the -1132/+277 Fugu GLUT4 promoter sequence. The three TSS are indicated with an asterisk (*). Positions are given relative to the major TSS assigned with the +1 position. The translation start codon ATG is indicated with capital letters. Putative binding sites for transcription factors are underlined. Promoter specific reverse GSP nested primer is underlined with an arrow. Blue boxes highlight the predicted CpG island.

**A single GLUT4 gene (slc2a4; ENSTRUG00000011935**)** was identified *insilico* in the Fugu genome sequence database. The Fugu GLUT4 gene maps to Scaffold_63 and its structure consists of 11 exons and 10 introns, spanning approximately 4.8 kb (** Figure **1A, B**)**. All exon-intron boundaries contained the consensus 5’- and 3’-splice donor and acceptor sequences, respectively (Table 1**)**. The translation initiation codon ATG and the termination codon TGA were located in exons 1 and 11, respectively. We determined the synteny between the human and the Fugu GLUT4 genes by identifying genes flanking the GLUT4 loci in the human and Fugu genomes (** Figure **1C**)**. Analysis of these loci identified several Fugu genes in Scaffold_63 (e.g. YBX2, EIF5AL1, GPS2, GABARAP, CTDNEP1, NEURL4, ACAP1**)** with homologs on human chromosome 17. The similarities between the Fugu and human GLUT4 loci with respect to the nature of the genes flanking the GLUT4 gene evidence a high degree of conservation in the organization of this important locus during evolution from fish to mammals.**

### Identification of the Fugu GLUT4 gene transcription start site (TSS)

We determined experimentally the position of the TSS of the Fugu GLUT4 gene by 5´RACE. This approach yielded three PCR product sizes of 452, 307 and 261 bp (Figure 1A). Analysis of these sequences demonstrated that the PCR products corresponded to three different transcripts starting at -145, +1 and +47, respectively. The abundant presence of the 307 bp transcript (Figure 1A), consistent with the observed basal activity of the different Fugu GLUT4 promoter deletion constructs (see below), led us to denote this as +1 and, consequently, as the main TSS of the GLUT4 gene in Fugu skeletal muscle.

### Cloning and sequence analysis of the Fugu GLUT4 *promoter*

**Figure 2:**
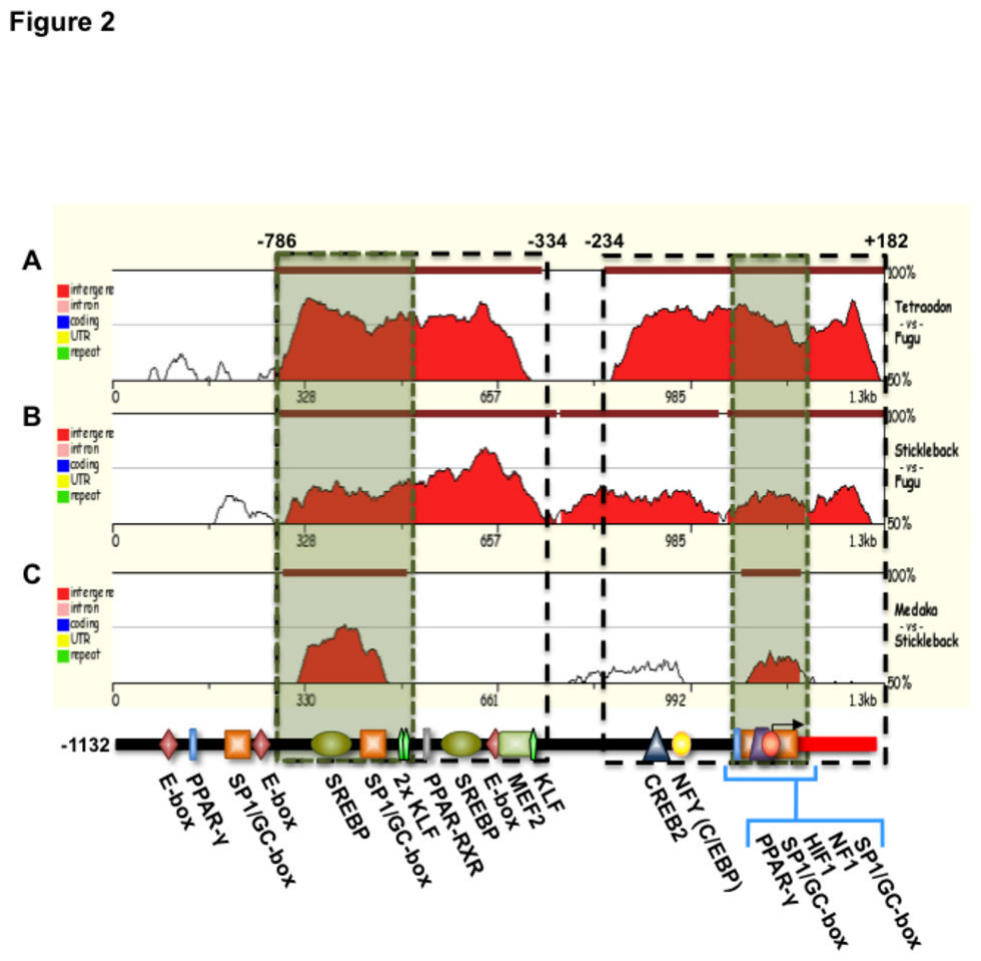
Conservation profile of the 5´ region of the known fish GLUT4 **genes**. Sequence elements of significant length (≥ 100 nucleotides) that present a sequence homology higher than 60% are highlighted in red and depicted with the dark-red rectangles on the top of the graph. Each graph represents the sequence comparison between the *Fugu* and *Tetraodon* (A), Fugu and Stickleback (B) and Stickleback and Medaka (C) GLUT4 promoters. The horizontal axis represents the position of the nucleotides within the 1314 bp sequence compared, starting at the 5’ end of the sequence. The vertical axis represents the percent of identity between the aligned genomes. In the bottom we show a schematic representation of the -1132 Fugu GLUT4 gene promoter that highlights the most relevant predicted binding sites. The open boxes delineating the regions comprised between -786/-334 and -234/+182 nucleotides represent conserved areas in teleost GLUT4 gene promoters.

To study the transcriptional regulation of the Fugu GLUT4 gene, we retrieved a 1.3 kb genomic sequence upstream of the GLUT4 gene from the Fugu genomic sequence database in Ensembl (Scaffold_63:258623-259996). Using sequence-specific primers (Table 1), a DNA fragment of 1,314 bp was amplified, starting 8 bp upstream of the Fugu GLUT4 ATG. *In silico* analysis of the cloned 1.3 kb 5´-flanking region of the Fugu GLUT4 gene revealed the presence of multiple putative binding sites for TFs such as MEF2, SREBP, KLF, SP1/GC-box, NF-Y, E-box, PPAR-γ, PPAR-RXR and HIF-1 (Figs. 1B and 2). However, this analysis also demonstrated that this region lacks identifiable TATA boxes. In this light, we searched for CpG islands that, together with the multiple predicted SP-1 transcription factor binding sites, are characteristic of TATA-less promoters [25,26]. Using MethPrimer software (http://www.urogene.org/methprimer/[17]) it was determined that the cloned genomic region of the Fugu GLUT4 gene contains a CpG island of 260 bases, between -234 and +93 nucleotides, with 163 CG dinucleotides (Figure 2).

Next, we examined the conservation profile of the 1.3 kb genomic DNA region upstream of the GLUT4 gene in teleost fish genomic sequences from available databases using zPicture software (Figure 2). Sequence comparison of this region between *Tetraodon* and Fugu showed two major regions, between -786/-334 and -234/+182 nucleotides, that were highly conserved, with a similarity higher than 70%, (Figure 2A). A similar conserved sequence profile was observed between stickleback and Fugu, with similarities between 50% and 70% in the conserved regions (Figure 2B). Despite the lack of significant similarities between the medaka and Fugu GLUT4 promoters, comparison between medaka and stickleback sequences confirmed that the GLUT4 promoters of all species analyzed contain two conserved areas, one of which surrounds the TSS+1 (Figure 2). Overall, these results indicate that the regions comprised between -786/-334 and -234/+182 nucleotides contain most of the relevant predicted binding motifs involved in the regulation of the Fugu GLUT4 gene, as well as the TSS+1.

### Functional identification of the transcriptional regulatory regions of the Fugu GLUT4 gene

**Figure 3:**
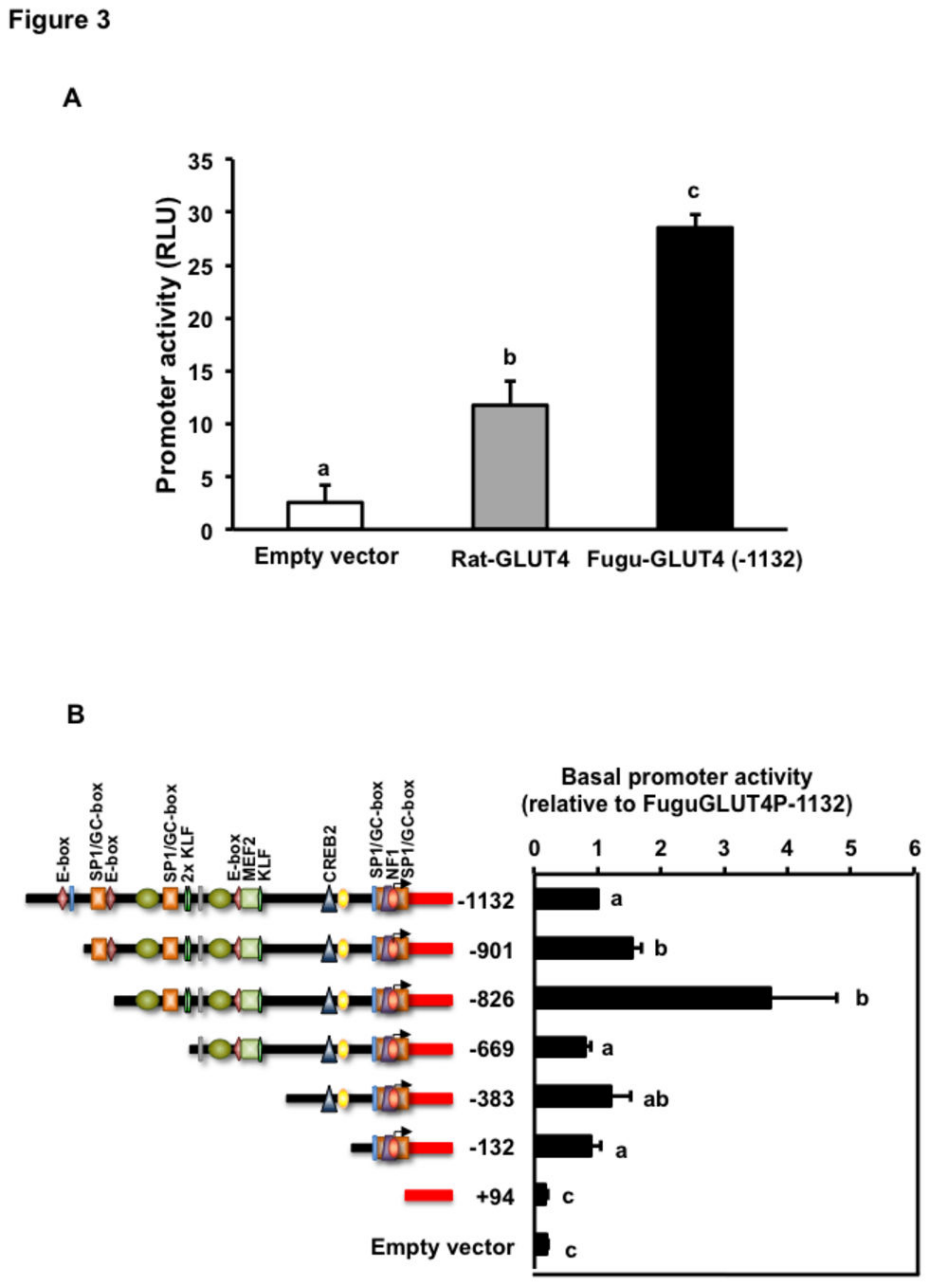
Basal activity of the Fugu GLUT4 promoter in transiently transfected L6 cells. (A) Basal activity of the empty vector (pGL3-Basic), rat GLUT4 (Rat-GLUT4) and Fugu GLUT4 (Fugu-GLUT4) promoters transiently transfected into L6 muscle cells. Data are shown as relative luciferase units (RLU) and expressed as mean ± S.E. of three independent experiments. (B) Basal activity of Fugu GLUT4 promoter deletion constructs transiently transfected into L6 muscle cells. Data on GLUT4 promoter activity are shown relative to that of the FuguGLUT4P-1132 promoter and expressed as mean ± S.E. of four independent experiments. Basal promoter activity of the FuguGLUT4P-1132 construct was 25.33 ± 3.4 (mean ± S.E.) RLU. Different letters indicate statistically significant differences (*p*<0.05).

To verify the functionality of the cloned Fugu GLUT4 promoter, we first transiently transfected the luciferase construct containing the 1.3 kb DNA fragment (FuguGLUT4P-1132) into L6 myoblasts. This cell line was chosen because of its extensive use in the study of GLUT4 biology in skeletal muscle and because of the lack of a suitable fish muscle cell line. In parallel, a similar luciferase construct containing 2.2 kb of the rat GLUT4 promoter was transiently transfected as a control for the activity of the cloned Fugu GLUT4 promoter. Our results indicated that the Fugu GLUT4 promoter was functional and that its basal activity was significantly higher than that of the rat GLUT4 promoter (Figure 3A).

In order to further characterize the promoter regions of the Fugu GLUT4 promoter that are responsible for its basal activity, we generated a set of six luciferase reporter gene constructs each containing serial deletions of the FuguGLUT4P-1132 construct (Figure 3B) and we transiently transfected them into L6 myoblasts. When compared to the parental FuguGLUT4P-1132 construct, the FuguGLUT4P-901 and FuguGLUT4P-826 constructs showed significantly higher transcriptional activity, whereas the activity of FuguGLUT4P-669, FuguGLUT4P-383 and FuguGLUT4P-132 constructs was similar to the parental FuguGLUT4P-1132 construct (Figure 3B). The FuguGLUT4P+94 construct presented similar levels of promoter activity than the empty vector (Figure 3B). The higher transcriptional activity of the FuguGLUT4P-901 and FuguGLUT4P-826 constructs suggests the presence of an upstream negative regulatory region, between -1132 and -901 nucleotides, and a positive regulatory region within this region, between -901 and -826 nucleotides. These results show that the region comprised between -132 and +94 contains essential elements required for the basal activity of the Fugu GLUT4 promoter. This is consistent with the *in silico* predictions that identified PPAR-γ, HIF1, SP1/GC-box and NF1 TF binding sites as well as a CpG island within this region where the TSS+1 is located. Therefore, these results strongly suggest that the core sequence of the basal promoter is localized in the region -132 to +94.

### Regulation of the activity of the Fugu GLUT4 promoter by insulin

**Figure 4:**
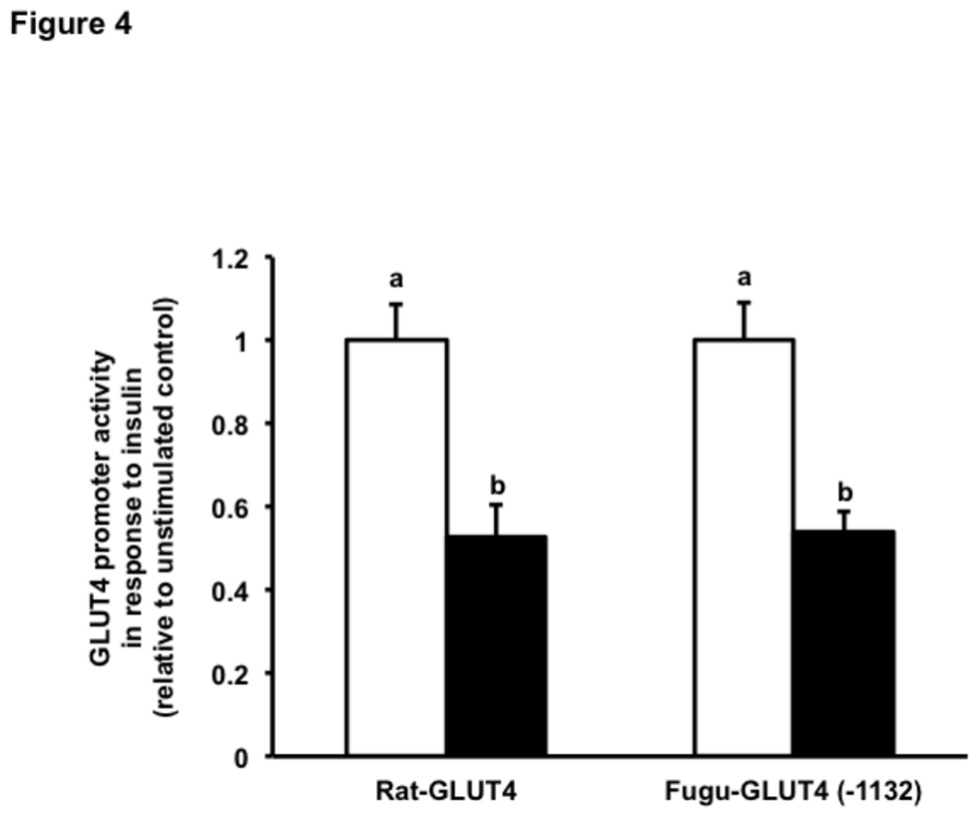
Activity of the rat and Fugu GLUT4 promoters in response to insulin. Rat and Fugu GLUT4 promoters were transiently transfected into L6 muscle cells and these were incubated in the absence (unstimulated control; white bars) or presence of human recombinant insulin (100 nM; black bars) for 18 hr. Data are normalized to the relative expression of Renilla luciferase activity, setting the activity of the unstimulated promoter to 1. Data on GLUT4 promoter activity in response to insulin are shown relative to that of the unstimulated control and expressed as mean ± S.E. of three independent experiments. Different letters indicate statistically significant differences (*p*<0.05).

**Figure 5:**
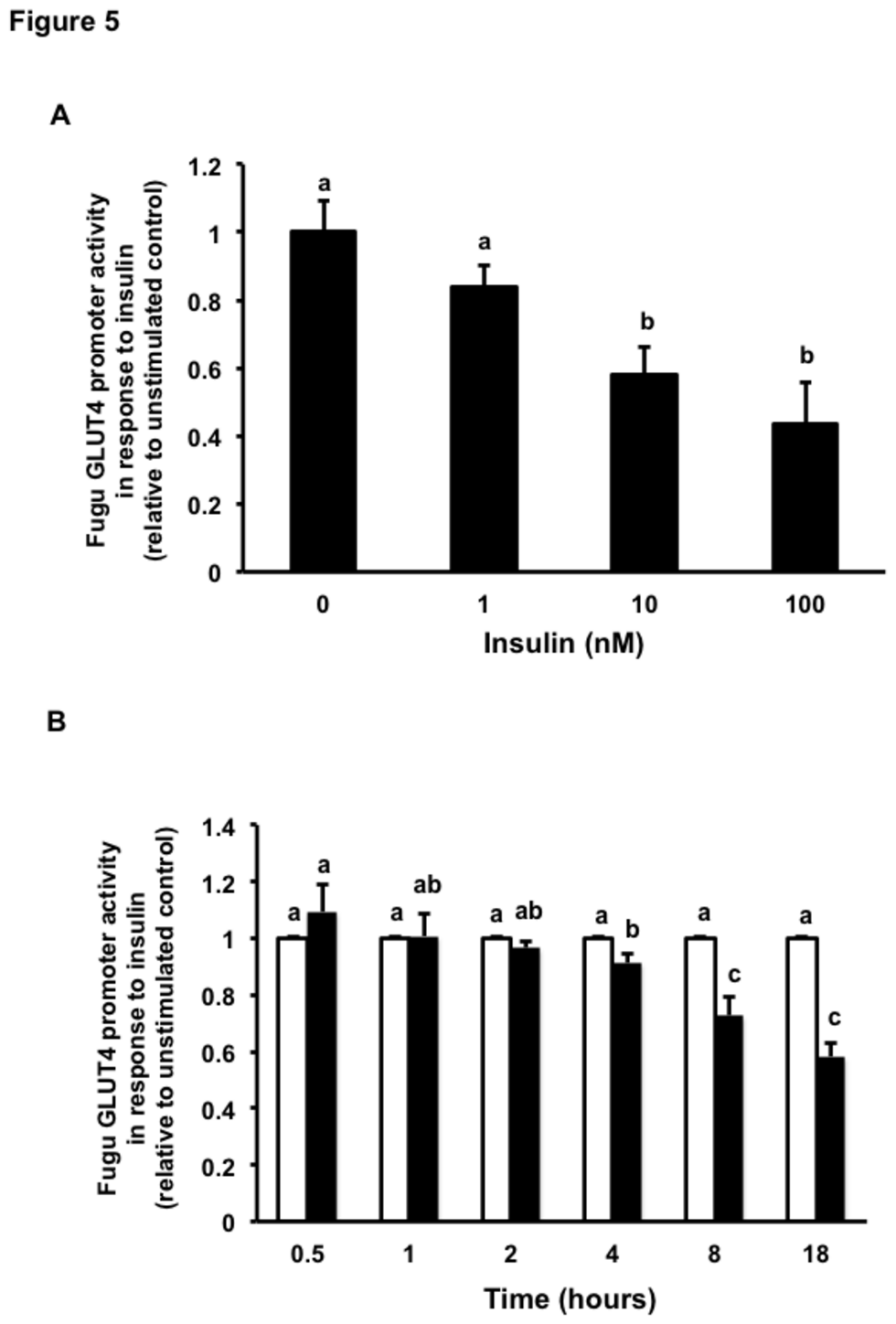
Dose- and time-dependent regulation of the activity of the Fugu GLUT4 promoter in response to insulin. (A) Dose-response effects of insulin. Transfected L6 muscle cells were incubated in the absence (unstimulated control) or presence of various concentrations of human recombinant insulin (1, 10 and 100 nM). Data are normalized to the relative expression of Renilla luciferase activity, setting the activity of the unstimulated promoter to 1. Data on Fugu GLUT4 promoter activity in response to insulin are shown relative to that of the unstimulated control and expressed as mean ± S.E. of four independent experiments. (B) Time-dependent effects of insulin. Transfected L6 muscle cells were incubated in the absence (unstimulated control; white bars) or presence of human recombinant insulin (100 nM; black bars) for for 0.5, 1, 2, 4, 8 and 18 hr. Data are normalized to the relative expression of Renilla luciferase activity, setting the activity of the unstimulated constructs to 1. Data on Fugu GLUT4 promoter activity in response to insulin are shown relative to that of the unstimulated control and expressed as mean ± S.E. of four independent experiments. Promoter activities of the unstimulated FuguGLUT4P-1132 construct at 0.5, 1, 2, 4, 8, 18 hrs were 22.82 ± 5.1; 34.27 ± 8.1; 26.31 ± 6.3; 20.61 ± 5.6; 34.61 ± 7.7 and 23.41 ± 3.2 (mean ± S.E.) RLU, respectively. Different letters indicate statistically significant differences (*p*<0.05).

In order to study whether insulin exerts a regulatory role on the expression of the Fugu GLUT4 gene at the level of transcription, we transiently transfected L6 myoblasts with the Fugu GLUT4 promoter or with the rat GLUT4 promoter, used as control, and stimulated transfected cells with human recombinant insulin (100 nM) for 18 h. Insulin treatment significantly decreased the activity of the Fugu and rat GLUT4 promoters by approximately 50% (Figure 4), confirming the previously described inhibitory effect of insulin on the rat GLUT4 promoter activity in L6 myoblasts and myotubes [22] as well as on the mouse GLUT4 promoter in 3T3-L1 adipocytes [27,28]. We further characterized the effects of insulin on the activity of the Fugu GLUT4 promoter by performing dose-response and time-course experiments in the absence or presence of insulin. Our results indicate that insulin significantly reduced the activity of the Fugu GLUT4 promoter in a dose- and time-dependent fashion. Specifically, the activity of the Fugu GLUT4 promoter was significantly reduced with respect to the control at 10 and 100 nM insulin (Figure 5A) and at 4, 8 and 18 h (Figure 5B) in the dose-response and time-course experiments, respectively. 

**Figure 6:**
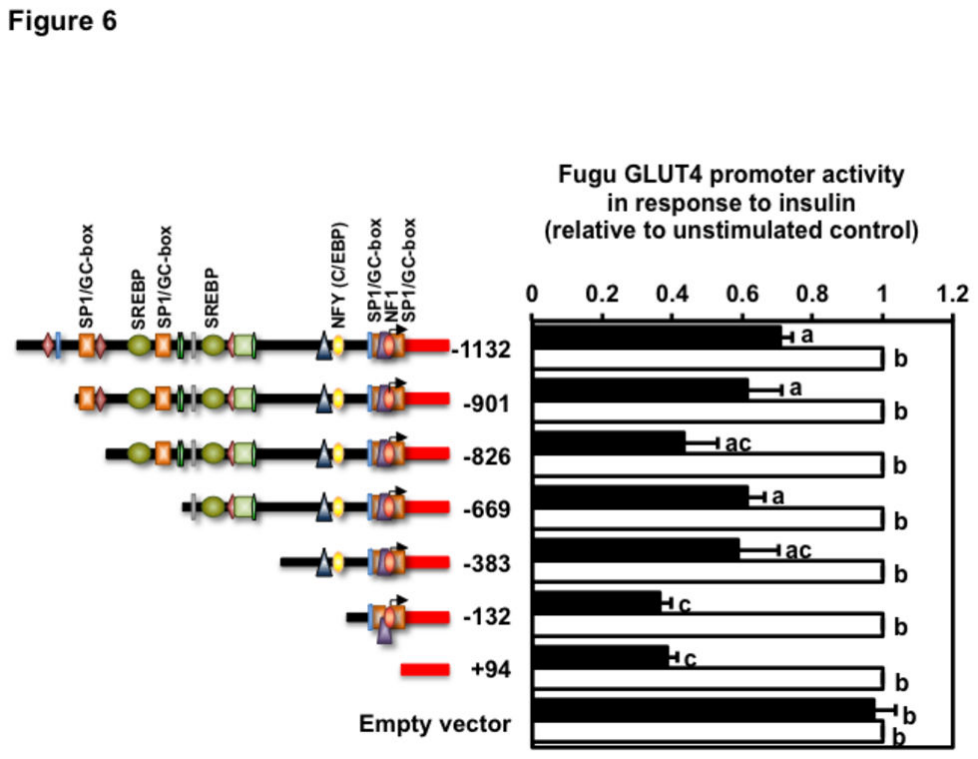
Activity of the Fugu GLUT4 promoter deletion constructs in response to insulin. L6 muscle cells transfected with the various constructs were incubated in the absence (unstimulated control) or presence of human recombinant insulin (100 nM) for 18 hr. Data are normalized to the relative expression of Renilla luciferase activity, setting the activity of the unstimulated constructs to 1. Data on the activity of the various promoter constructs in response to insulin are shown relative to that of the unstimulated control constructs and expressed as mean ± S.E. of four independent experiments. Basal promoter activity of the FuguGLUT4P-1132 construct was 23.3 ± 2.8 (mean ± S.E.) RLU. Different letters indicate statistically significant differences (*p*<0.05).

Next, we set out to identify the regions potentially involved in the regulation of the activity of the Fugu GLUT4 promoter by insulin. For this purpose, we transiently transfected rat L6 myoblasts with the various deletion constructs and incubated the cells for 18 h in the absence or presence of insulin (100 nM). L6 myoblasts transiently transfected with any of the six deletion constructs showed a significant reduction in their transcriptional activity in response to insulin (Figure 6). The two shorter constructs (i.e. FuguGLUT4P-132 and FuguGLUT4P+94) showed the lowest level of activity in response to insulin.

### Regulation of the activity of the Fugu GLUT4 promoter by PPARγ

**Figure 7:**
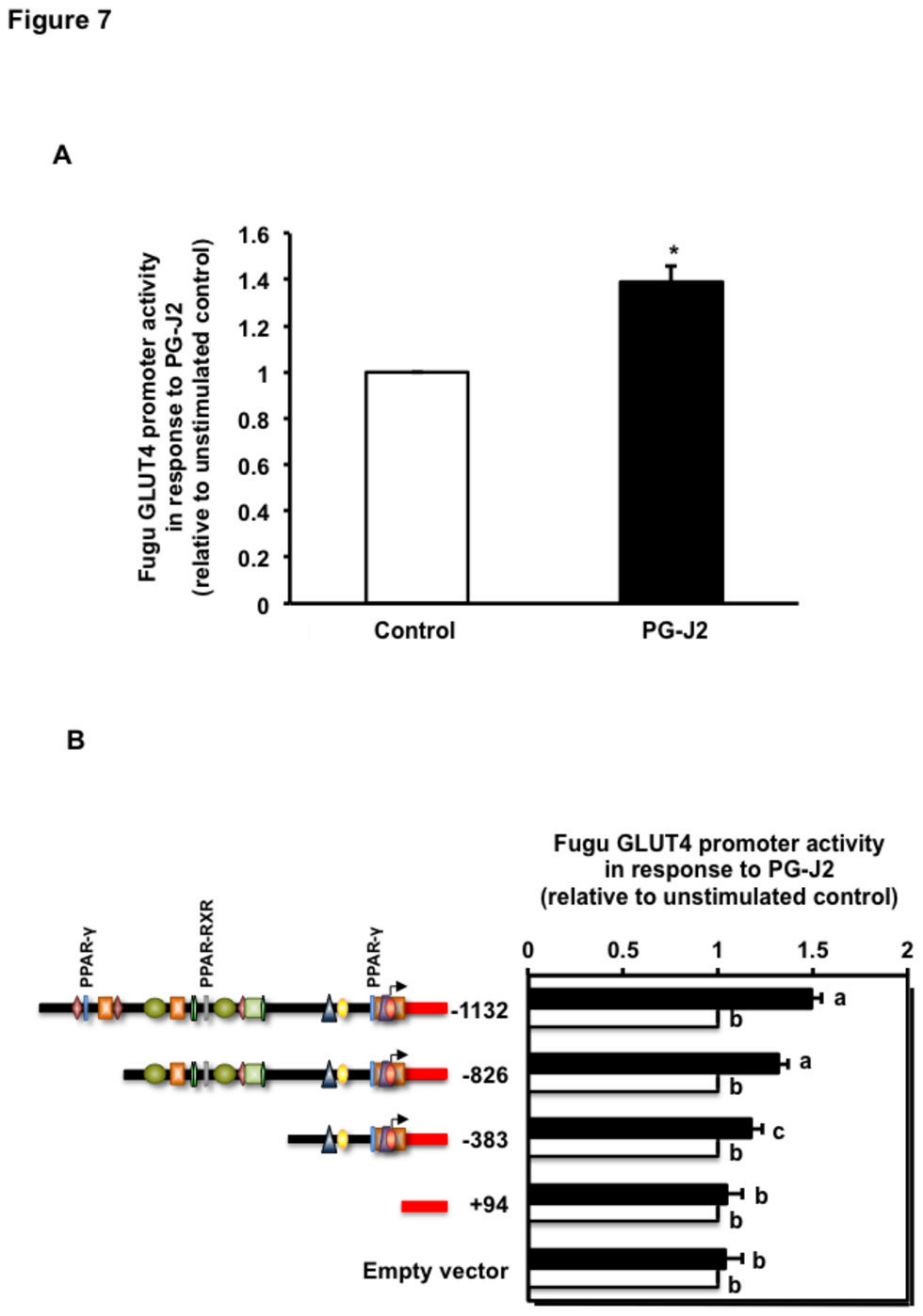
Activity of the Fugu GLUT4 promoter in response to ligand-induced PPARγ activation. (A) Effects of PG-J2 on the Fugu GLUT4 promoter activity. The -1132 Fugu GLUT4 promoter construct was transiently transfected into L6 muscle cells and stimulated with PG-J2 (10 μM) for 18 hr. Data on Fugu GLUT4 promoter activity in response to PG-J2 are shown relative to that of the unstimulated control and expressed as mean ± S.E. of three independent experiments. * indicates statistically significant differences (*p*<0.05). (B) Activity of Fugu GLUT4 promoter deletion constructs in response to stimulation with PG-J2. Data are normalized to the relative expression of Renilla luciferase activity, setting the activity of the unstimulated constructs to 1. Data on the activity of the various promoter constructs in response to PG-J2 are shown relative to that of the unstimulated control constructs and expressed as mean ± S.E. of three independent experiments. Different letters indicate statistically significant differences (*p*<0.05). In graphs A and B, the activity of the unstimulated FuguGLUT4P-1132 construct was 22.57 ±0.36 (mean ± S.E.) RLU.

Given the presence of putative binding sites for PPARs (PPARγ and PPAR-RXR) in the Fugu GLUT4 promoter sequence, we next asked whether PPARγ could be involved in the regulation of the Fugu GLUT4 gene at the transcriptional level by testing the effects of PG-J2, a natural PPARγ ligand. For this purpose, we stimulated L6 myoblasts that were transiently transfected with the FuguGLUT4P-1132 construct in the absence or presence of PG-J2 (10 μM) for 18 h. Our results show that treatment with PG-J2 significantly increased the transcriptional activity of the Fugu GLUT4 promoter (Figure 7A). To identify the regions involved in the regulation of the Fugu GLUT4 gene transcription by the PPARγ ligand, we transiently transfected L6 myoblasts with the FuguGLUT4P-826, -383 and +94 deletion constructs and incubated cells in the absence or presence of PG-J2 (10 μM) for 18 h. L6 myoblasts expressing the FuguGLUT4P-826 and -383 constructs showed a significant increase in their transcriptional activity in response to PG-J2 whereas cells expressing the +94 deletion construct did not show any change in response to PG-J2 (Figure 7B). Interestingly, we observed a relationship between the number of PPARγ binding motifs present in Fugu GLUT4 promoter and its response to PG-J2. Sequential deletions of the PPARγ binding motifs in the parental GLUT4 promoter induced a progressive reduction of the stimulatory effects of PG-J2, as shown by the significant reduction of the activity of the FuguGLUT4P-383 construct when compared with that of the FuguGLUT4P-1132 (parental) construct (Figure 7B).

### Regulation of the activity of the Fugu GLUT4 promoter by electrical pulse stimulation

**Figure 8:**
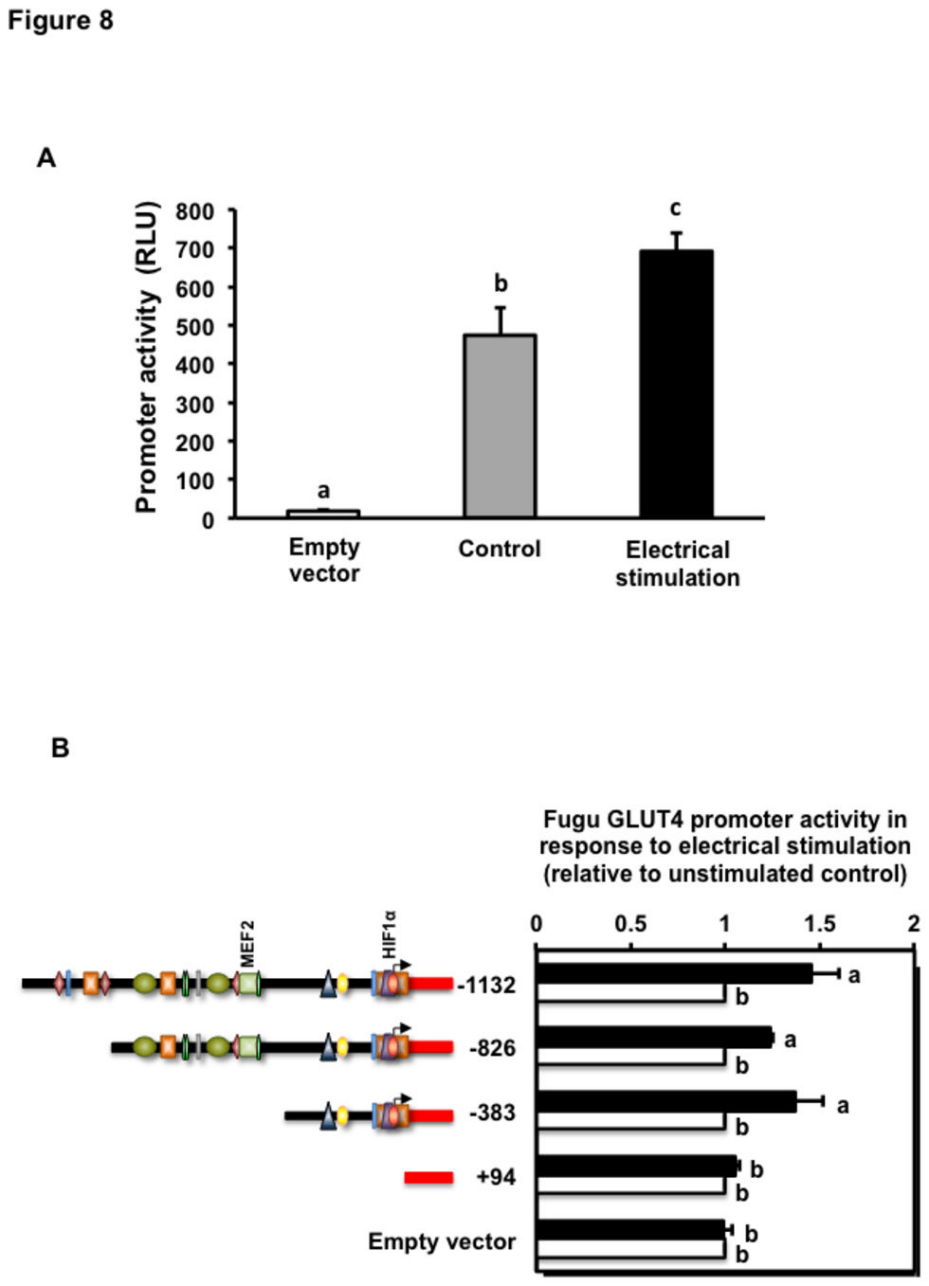
Activity of the Fugu GLUT4 promoter in response to *in* *vitro* electrical pulse stimulation. (A) Effects of electrical pulse stimulation on the Fugu GLUT4 promoter activity. The -1132 Fugu GLUT4 promoter construct was transiently transfected into C2C12 muscle cells and electrically stimulated as described in Materials and Methods. Data are shown as relative luciferase units (RLU) and are expressed as mean ± S.E. of four independent experiments. Different letters indicate statistically significant differences (*p*<0.05). (B) Activity of Fugu GLUT4 promoter deletion constructs in response to electrical pulse stimulation. Data are normalized to the relative expression of Renilla luciferase activity, setting the activity of the unstimulated construct to 1. Data on the activity of the various promoter constructs in response to electrical stimulation are shown relative to that of the unstimulated control constructs and expressed as mean ± S.E. of three independent experiments. Promoter activity of the unstimulated FuguGLUT4P-1132 construct was 416.7 ±82.7 (mean ± S.E.) RLU. Different letters indicate statistically significant differences (*p*<0.05).

We next asked whether contractile activity could regulate the transcriptional activity of the Fugu GLUT4 gene in mouse C2C12 myotubes. *In vitro* differentiated mouse C2C12 myotubes, contrary to L6 myotubes, develop a contractile apparatus of sarcomere units and are able to contract in response to electrical pulse [23,29] and cholinergic stimulation [30]. Therefore, we used this established cell line to investigate the regulation of the activity of the Fugu GLUT4 promoter by contractile activity as induced by electrical pulse stimulation. Our results show that C2C12 myotubes expressing the FuguGLUT4P-1132 construct that were stimulated electrically had significantly higher levels of luciferase activity than non-stimulated cells (Figure 8A). Additionally, in order to characterize regions potentially involved in the induction of the Fugu GLUT4 gene transcription by contractile activity, we studied its effects on C2C12 myotubes transiently transfected with the -826, -383 and +94 deletion constructs. Interestingly, the activity of the FuguGLUT4P-826 and -383 deletion constructs was significantly up-regulated by electrical stimulation, while the +94 deletion construct showed no changes in its transcriptional activity (Figure 8B).

## Discussion

The GLUT4 gene is widely considered essential for the control of glucose homeostasis in mammals. Although the GLUT4 gene is present in the sequenced genomes of most non-mammalian vertebrate species, no information is available on its characterization and its transcriptional regulation outside of mammals. Given our previous studies on the regulation of the expression of the GLUT4 gene at the mRNA and protein levels in teleost fish [14,15,31-33], we set out to identify and characterize the GLUT4 gene in Fugu, a teleost model species with a fully sequenced genome, with emphasis on its regulatory region and its control. In the present study we show that the Fugu GLUT4 gene has a similar structure as the human and mouse GLUT4 genes, with 11 exons and 10 introns, and also a similar gene arrangement around the GLUT4 gene locus. This evidences a remarkable degree of conservation in the structure and location of the GLUT4 gene during vertebrate evolution. Importantly, in the present study, we have also identified and characterized the first functional GLUT4 promoter in a non-mammalian species. The 5´-flanking region of the Fugu GLUT4 gene contains 3 TSSs, a conserved cluster of CpG dinucleotides, three SP1 binding motifs and lacks TATA-box elements. These findings are consistent with previous reports indicating that GC-rich promoter regions are typically characterized by the presence of multiple TSSs, the lack of TATA elements and the presence of multiple SP1 transcription factor binding sites [25,26.34,35]. Interestingly, the structure of the Fugu GLUT4 gene promoter correlates well with that of mammalian GLUT4 promoters that also present multiple TSSs, lack TATA-box elements, contain binding sites for several nuclear transcription factors including SP1 and C/EBP and present GC-rich regions close to the core promoter [36-39]. By comparing the structure of the cloned Fugu GLUT4 promoter with that of other teleost species, we have identified two highly conserved regions that contain most of the binding motifs potentially involved in the transcriptional regulation of the GLUT4 gene (Figure 2). Within these regions, the E-box/MEF2/Klf cassette, located between nucleotides -531 and -478, and the core promoter appear strongly conserved in all the species analyzed (Figure 2). Similarly, in the rat, mouse and human GLUT4 promoters there is a highly conserved region containing this cassette as part of a well-characterized enhancer region [12]. It is known that the Klf15 and E-box binding factors exert a synergistic effect on MEF2 [12], which is an essential binding site for the tissue-specific expression of the GLUT4 gene in mammals [10,11,40]. The presence of CG-rich regions in the Fugu as in mammalian GLUT4 promoters and the known potential methylation of the latter [41], strongly suggests that the Fugu GLUT4 gene could be subjected to epigenetic regulation. Given the extreme sensitivity of ectothermic aquatic vertebrates to environmental changes, future studies should aim at investigating the potential epigenetic regulation of the GLUT4 gene in teleosts.

From a functional point of view, we have demonstrated that the Fugu GLUT4 promoter is active when expressed in the rat muscle cell line L6. Furthermore, by generating a series of 5´-deletion constructs of the Fugu GLUT4 upstream regulatory region, we have determined that the +1 TSS as well as the presumptive core promoter are indeed located between nucleotides -132 and +93, matching completely the position of the predicted CpG island. The -901 and -826 constructs were the only deletions showing a significant increase in basal activity over that of the -1132 promoter construct. This finding suggests the existence of an enhancer region between nucleotides -901 and -669, as also supported by the presence of a tandem of binding sites for Klf15 between nucleotides -665 and -677. In mammals, Klf15 strongly induces GLUT4 transcription by synergizing with MEF2 [42]. 

Insulin is an effective inducer of GLUT4 mRNA and protein expression as well as of GLUT4 translocation in fish [14,15,31-33] as in mammals [43-45]. Strikingly, the effect of insulin on the regulation of the transcription of the GLUT4 gene has not been fully characterized to date, even in mammals. In fact, several published observations indicate that insulin may exert a paradoxical inhibitory effect on the transcription of the GLUT4 gene. Cooke and Lane observed that insulin caused a repression of GLUT4 transcription that was mediated by NF1 in 3T3L1 adipocytes [27]. More recently, insulin was shown to inhibit GLUT4 transcription in mammalian L6 muscle cells [22]. In the present study, we have confirmed the insulin-induced repression of the rat GLUT4 promoter, used as a control for the activity of the Fugu GLUT4 promoter. Interestingly, we have also observed that the activity of the Fugu GLUT4 promoter is inhibited by insulin in a dose- and time-dependent manner. The inhibition of the transcriptional activity of the fish and mammalian GLUT4 promoters by insulin is difficult to reconcile with the known stimulatory effects of insulin on GLUT4 mRNA levels in skeletal muscle in fish and mammalian species. Several possibilities could be offered to try to explain this discrepancy. First, it is possible that the regions in the GLUT4 promoter that may mediate its transcriptional activation by insulin may have been located upstream of the cloned GLUT4 promoters examined (i.e. 1.3 kb for the Fugu gene and 2.2 kb for the rat gene). Second, it is possible that prolonged treatment of L6 muscle cells in the presence of insulin may have induced insulin resistance. However, the minimal effective insulin concentrations (10 nM) and incubation times with insulin (4 h) that lead to the decrease in the activity of the Fugu GLUT4 promoter are difficult to reconcile with the idea that insulin may be causing an insulin resistance phenotype in L6 cells, although we cannot completely rule out the possibility that insulin treatment in transfected L6 cells may have caused a non-physiological effect on GLUT4 promoter activity. Interestingly, in the case of the Fugu GLUT4 promoter, all the promoter deletions, including the +94 construct, were repressed by insulin, suggesting that the promoter region that is downstream of the TSS may contain the necessary elements for mediating the repression of the GLUT4 gene by insulin. Clearly, further studies will be needed to identify the regulatory regions responsible for the activation of vertebrate GLUT4 promoters by insulin.

In order to study other mechanisms potentially involved in the regulation of the Fugu GLUT4 promoter activity, we first investigated the *in vitro* effects of PPARγ activation. PPARs are ligand–activated transcription factors from the nuclear receptor family. Three PPAR isoforms (α, β and γ) have been described, and they differ in their tissue distribution and ligand specificity [46]. In particular, PPARγ is involved in the regulation of lipid metabolism and glucose homeostasis [47] and is expressed in adipose and muscle tissue [48]. Furthermore, PPARγ has been shown to repress GLUT4 promoter activity in adipocytes [49], while treatment with synthetic PPARγ agonist agents called thiazolidinediones (TZD) in obese Zucker fa/fa rats increased GLUT4 mRNA levels in adipose tissue [50]. It is suggested that unbound PPARγ represses GLUT4 transcription and that PPARγ ligands alleviate the repression, consequently increasing GLUT4 expression [13]. PG-J2 has been shown to be the most potent natural ligand of PPARγ [51,52]. Our results demonstrate that transcription of the Fugu GLUT4 gene is significantly induced by PG-J2, decreasing gradually with the ablation of the PPAR/RXR motifs. This is consistent with the fact that PPARγ receptors heterodimerize with retinoid X receptor-α (RXR) to exert their transcriptional activation [49]. Although our results strongly suggest that the Fugu GLUT4 promoter is induced by PPARγ activation when expressed in mammalian muscle cells, it is not known if the fish GLUT4 gene can be activated by PPARγ in skeletal muscle in vivo. Like in mammals, the basal expression of PPARγ in fish skeletal muscle is very low in a number of fish species examined to date [53-56], including in Fugu [57]. Furthermore, fish PPARγ is known to be modestly sensitive [55,58,59] or not sensitive at all [60] to PPARγ ligands such as PG-J2 and rosiglitazone. Further studies evaluating the effects of PPARγ ligands in fish skeletal muscle using vitro and in vivo models are needed to resolve to potential physiological significance of the results of the present study. 

Muscle contraction and chronic contractile activity of skeletal muscle cells have also been reported to stimulate GLUT4 gene transcription [61-64]. In the present study, we have used electrical pulse stimulation in differentiated C2C12 myotubes to mimic the effects of exercise *in vitro*. Our results clearly show that electrical stimulation of skeletal muscle cells results in an increase in Fugu GLUT4 gene transcription. Interestingly, all the deletion constructs except the one lacking the HIF-1 motif (+94) showed a significant increase in the transcriptional activity in response to electrical pulse stimulation. This result is in agreement with previous data indicating that HIF-1 binding factor participates in contraction-induced GLUT4 transcriptional activity [62,63]. Furthermore, these results support the notion that GLUT4 expression is induced under hypoxic conditions [65] and provide new evidence for the important role of HIF-1 as an activator of the transcription of the GLUT4 gene when the partial tension of oxygen falls in muscle fibers during exercise [66]. Our group recently reported for the first time that swimming-induced exercise in rainbow trout increases GLUT4 mRNA levels in skeletal muscle [16] and hypothesized that the known stimulatory effects of swimming on glucose metabolism in fish [67] could be mediated, at least in part, by increased expression of the GLUT4 gene. The results from the present study strongly suggest that the mechanisms by which swimming causes increased GLUT4 expression in fish skeletal muscle and, consequently, increased glucose entry and utilization in this tissue could involve the transcriptional activation of the GLUT4 gene. 

In summary, in the present study we have cloned and characterized the first functional GLUT4 gene promoter in a non-mammalian species. The Fugu GLUT4 gene promoter presents most of the binding sites described as important for the transcriptional regulation of the mammalian GLUT4 gene. Moreover, in agreement with data available in mammals, the Fugu GLUT4 gene promoter is regulated by insulin, PPARγ and contractile activity, suggesting evolutionarily conserved mechanisms leading to the regulation of the expression of the main insulin-regulated glucose transporter, GLUT4. Given that insulin also stimulates the translocation of the GLUT4 protein to the plasma membrane in fish skeletal muscle cells [15,68], as in mammals, the information available to date indicates that the various molecular and cellular mechanisms regulating the amount of GLUT4 present in the cell surface have been fairly well conserved during vertebrate evolution. However, important differences between fish and mammalian GLUT4 identified to date reside in their intracellular traffic characteristics [15,68,69] and in the lower affinity for glucose of fish GLUT4 [31], differences that have been associated with the known lower ability of fish, when compared to mammals, to regulate glucose levels in the blood.

## Supporting Information

Figure S1 **Genomic structure and chromosomal localization of the Fugu GLUT4 (slc2a4) gene.** (A) Organization of the Fugu GLUT4 gene. Exons are numbered and indicated by boxes and introns are indicated by lines. (B) Position of the genomic region containing the human GLUT4 gene (indicated by a red box) in human chromosome 17 (HsCh.17) (top) and localization of the Fugu GLUT4 gene in Scaffold_63, as indicated by a red box (bottom). (C) Synteny of the regions containing the GLUT4 gene in HsCh.17 and Fugu Scaffold_63. Surrounding *slc2a4*, the genes *ybx2* (Y box binding protein 2), *eif5a* (eukaryotic translation initiation factor 5A), *gps2* (G protein pathway suppressor 2), *neurl4* (neuralized homolog 4), *acap1* (ArfGAP with coiled-coil, ankyrin repeat and PH domains 1), *ctdnep1* (CTD nuclear envelope phosphatase 1) and *gabarap* (GABA(A) receptor-associated protein) appear in both species but arranged in slightly different order.
(TIF)

Figure S2 **Prediction of a CpG island within the 5´ cloned region of the Fugu GLUT4** **gene**. Black arrows indicate the positions delimiting the CpG island relative to the +1 TSS. Blue area indicates the position of the CpG island.
(TIF)
