# Correction: Mechanisms Regulating GLUT4 Transcription in Skeletal Muscle Cells Are Highly Conserved across Vertebrates

**DOI:** 10.1371/annotation/93141e7a-61f3-48bd-87bd-216b030d773d

**Published:** 2014-01-07

**Authors:** Rubén Marín-Juez, Mónica Diaz, Jordi Morata, Josep V. Planas

There are several errors in the Primer Name and Usage columns of Table 1. Please see the corrected Table 1 here: 

**Figure:**
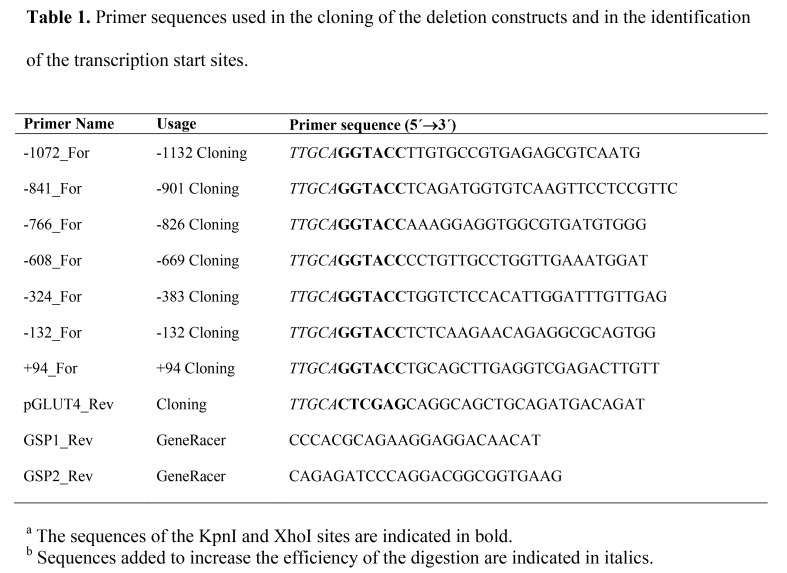


In the first paragraph of the Results section, the three figure references are incorrect. 

-The second sentence should refer to Supplementary Figures 1A, B. The correct sentence is: "The Fugu GLUT4 gene maps to Scaffold_63 and its structure consists of 11 exons and 10 introns, spanning approximately 4.8 kb (Supplementary Figures 1A, B)."

-The third sentence should refer to Supplementary Table 1, which is included as a part of this correction. The correct sentence is: "All exon-intron boundaries contained the consensus 5’- and 3’-splice donor and acceptor sequences, respectively (Supplementary Table 1)."

-The fifth sentence should refer to Supplementary Figure 1C. The correct sentence is: "We determined the synteny between the human and the Fugu GLUT4 genes by identifying genes flanking the GLUT4 loci in the human and Fugu genomes (Supplementary Figure 1C)."

The Figure 6 legend is missing a key for the right-hand graph. The unstimulated control should be associated with the white bars in the graph, and the human recombinant insulin (100 nM) with the black bars. The corrected figure legend is as follows:

"L6 muscle cells transfected with the various constructs were incubated in the absence (unstimulated control; white bars) or presence of human recombinant insulin (100 nM; black bars) for 18 hr. Data are normalized to the relative expression of Renilla luciferase activity, setting the activity of the unstimulated constructs to 1. Data on the activity of the various promoter constructs in response to insulin are shown relative to that of the unstimulated control constructs and expressed as mean ± S.E. of four independent experiments. Basal promoter activity of the FuguGLUT4P-1132 construct was 23.3 ± 2.8 (mean ± S.E.) RLU. Different letters indicate statistically significant differences (p<0.05)."

Section B of the Figure 7 legend is missing a key for the lower right-hand graph. The PG-J2 should be associated with the black bars in the graph, and the unstimulated control constructs with the white bars. The corrected figure legend reads: 

"(A) Effects of PG-J2 on the Fugu GLUT4 promoter activity. The -1132 Fugu GLUT4 promoter construct was transiently transfected into L6 muscle cells and stimulated with PG-J2 (10 μM) for 18 hr. Data on Fugu GLUT4 promoter activity in response to PG-J2 are shown relative to that of the unstimulated control and expressed as mean ± S.E. of three independent experiments. * indicates statistically significant differences (p<0.05). (B) Activity of Fugu GLUT4 promoter deletion constructs in response to stimulation with PG-J2. Data are normalized to the relative expression of Renilla luciferase activity, setting the activity of the unstimulated constructs to 1. Data on the activity of the various promoter constructs in response to PG-J2 (black bars) are shown relative to that of the unstimulated control constructs (white bars) and expressed as mean ± S.E. of three independent experiments. Different letters indicate statistically significant differences (p<0.05). In graphs A and B, the activity of the unstimulated FuguGLUT4P-1132 construct was 22.57 ±0.36 (mean ± S.E.) RLU."

Section B of the Figure 8 legend is missing a key for the right-hand graph. The first sentence should be associated with the black bars in the graph, and the unstimulated construct mentioned in the second sentence with the white bars. The corrected figure legend reads:

"(A) Effects of electrical pulse stimulation on the Fugu GLUT4 promoter activity. The -1132 Fugu GLUT4 promoter construct was transiently transfected into C2C12 muscle cells and electrically stimulated as described in Materials and Methods. Data are shown as relative luciferase units (RLU) and are expressed as mean ± S.E. of four independent experiments. Different letters indicate statistically significant differences (p<0.05). (B) Activity of Fugu GLUT4 promoter deletion constructs in response to electrical pulse stimulation (black bars). Data are normalized to the relative expression of Renilla luciferase activity, setting the activity of the unstimulated construct to 1 (white bars). Data on the activity of the various promoter constructs in response to electrical stimulation are shown relative to that of the unstimulated control constructs and expressed as mean ± S.E. of three independent experiments. Promoter activity of the unstimulated FuguGLUT4P-1132 construct was 416.7 ±82.7 (mean ± S.E.) RLU. Different letters indicate statistically significant differences (p<0.05)."

Supplementary Table 1 is missing. Please find a copy of the table here: 

Download corrected item

