# Supplementary figures and images for: Mechanisms Regulating GLUT4 Transcription in Skeletal Muscle Cells Are Highly Conserved across Vertebrates
Source: PLoS One. 2013 Nov 18;8(11):e80628. doi: 10.1371/journal.pone.0080628 (PMC3832493; doi:10.1371/journal.pone.0080628)

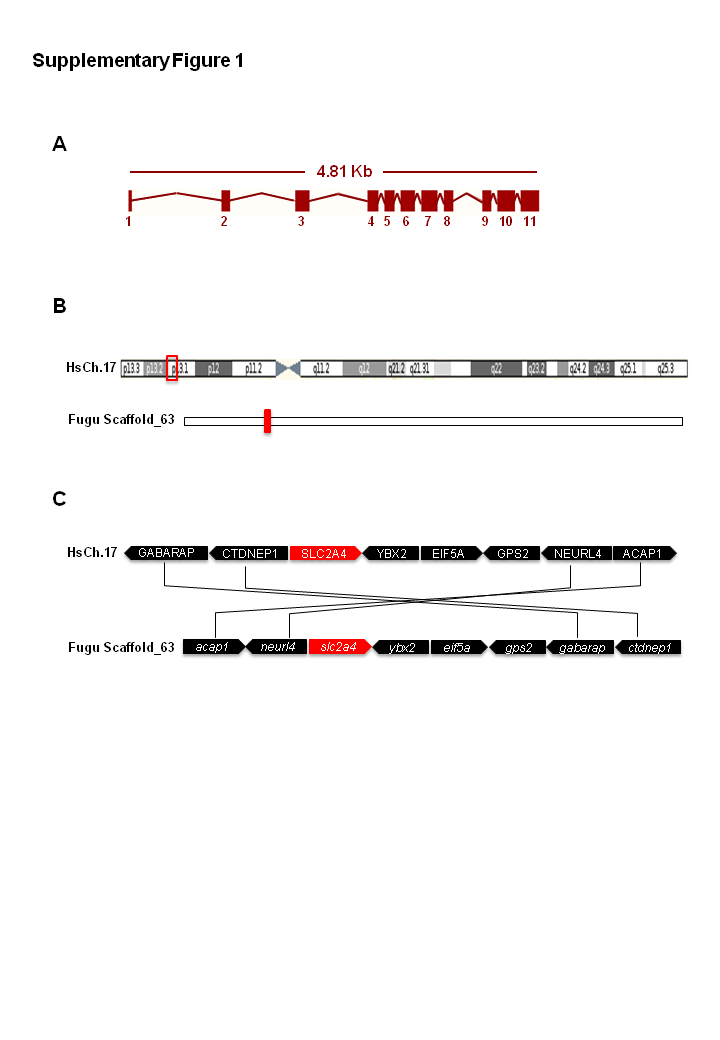

Supplement: Figure S1 — Genomic structure and chromosomal localization of the Fugu GLUT4 (slc2a4) gene. (A) Organization of the Fugu GLUT4 gene. Exons are numbered and indicated by boxes and introns are indicated by lines. (B) Position of the genomic region containing the human GLUT4 gene (indicated by a red box) in human chromosome 17 (HsCh.17) (top) and localization of the Fugu GLUT4 gene in Scaffold_63, as indicated by a red box (bottom). (C) Synteny of the regions containing the GLUT4 gene in HsCh.17 and Fugu Scaffold_63. Surrounding slc2a4, the genes ybx2 (Y box binding protein 2), eif5a (eukaryotic translation initiation factor 5A), gps2 (G protein pathway suppressor 2), neurl4 (neuralized homolog 4), acap1 (ArfGAP with coiled-coil, ankyrin repeat and PH domains 1), ctdnep1 (CTD nuclear envelope phosphatase 1) and gabarap (GABA(A) receptor-associated protein) appear in both species but arranged in slightly different order. (TIF) [file pone.0080628.s001.tif]

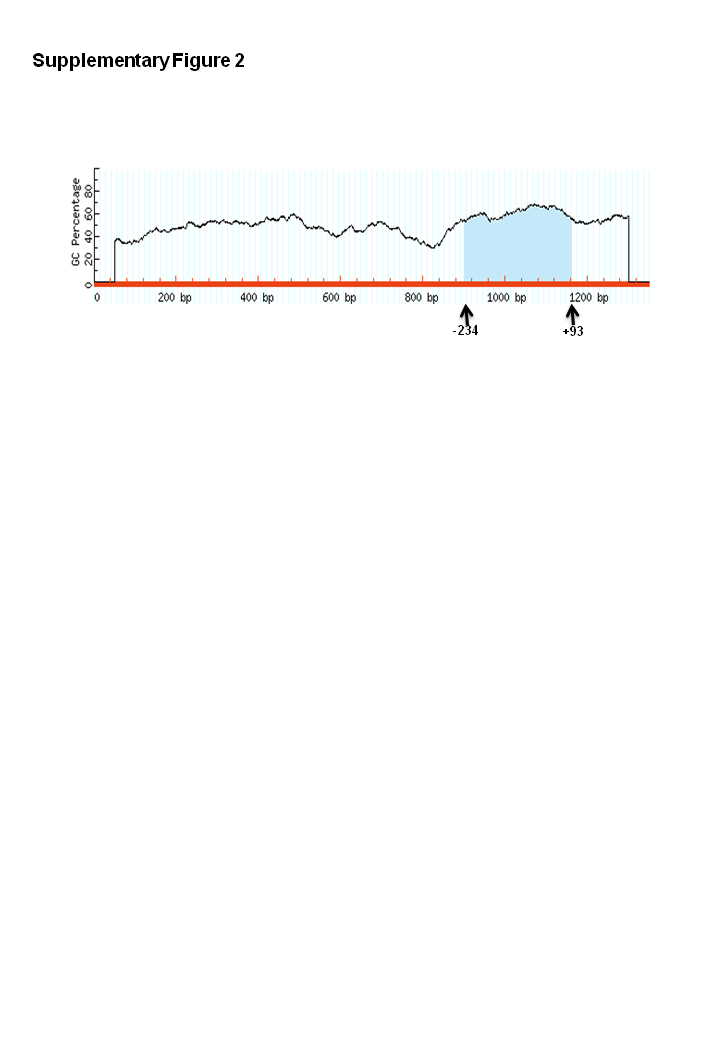

Supplement: Figure S2 — Prediction of a CpG island within the 5´ cloned region of the Fugu GLUT4 gene. Black arrows indicate the positions delimiting the CpG island relative to the +1 TSS. Blue area indicates the position of the CpG island. (TIF) [file pone.0080628.s002.tif]
