# Supplementary material for: Correction: Mechanisms Regulating GLUT4 Transcription in Skeletal Muscle Cells Are Highly Conserved across Vertebrates
Source: PLoS One. 2014 Jan 7;9(1):10.1371/annotation/93141e7a-61f3-48bd-87bd-216b030d773d. doi: 10.1371/annotation/93141e7a-61f3-48bd-87bd-216b030d773d (PMC6061768; doi:10.1371/annotation/93141e7a-61f3-48bd-87bd-216b030d773d)
Supplement: Download corrected item [file 93141e7a-61f3-48bd-87bd-216b030d773d.s001.pdf]

Supplementary Table 1. Exon-intron organization of the Fugu GLUT4 gene

[a] Exon and intron sequences are indicated in upper and lower-case letters, respectively.

				**Sequence at Exon-Intron Junction**				
**Exon No.**	**Exon Start**	**Exon End**	**Exon Size (bp)**	**Exon Sequence (3')**	**5´-Splice donor**	**Intron Size (bp)**	**3'-Splice Acceptor**	**Exon Sequence (5')**
1	258,674	258,642	33	GGGGAG	gtattaattt	1,056	tgttttcag	ACAGTG
2	257,585	257,490	96	CAGAAG	gtagagag	768	ctctgcag	AGGATC
3	256,721	256,561	161	GGGCAG	gtagtggac	683	catttccag	GAGAAA
4	255,877	255,753	125	ACTGTG	gcgagtgc	73	ctgcgcag	GACTTG
5	255,679	255,564	116	GCACAG	gtaccacagc	76	ctgttaag	ATCCTG
6	255,487	255,325	163	AGAGAG	gtgagggt	77	ttttcgcag	GTCTAA
7	255,247	255,060	188	AACGCA	gtgagtccc	78	ctggacag	ATTTTC
8	254,981	254,877	105	GTCTCG	gtaaccaaa	345	tcccaacag	CTCTTC
9	254,531	254,430	102	TTGCTG	gtcagtctcg	72	gtacactag	GACAGT
10	254,357	254,154	204	GTCGCG	gtaaggatt	69	ccgtcctcag	AACCTC
11	254,084	253,869	216	AACTGA				
